# Generalization of finger-joint kinematics for cleaning tasks

**DOI:** 10.3389/frobt.2026.1725261

**Published:** 2026-02-24

**Authors:** Clara Pham, Jan-Philipp Tauscher, Colin Groth, Jochen J. Steil

**Affiliations:** 1 Institute for Robotics and Process Control, Technische Universität Braunschweig, Braunschweig, Germany; 2 Institute for Computer Graphics, Technische Universität Braunschweig, Braunschweig, Germany; 3 Immersive Computing Lab, New York University Tandon School of Engineering, New York, NY, United States

**Keywords:** dimensionality reduction, kinematics, movement primitives, multi-finger, robotic hand, simulation

## Abstract

Achieving robust, dexterous manipulation in unstructured environments remains a central challenge in robotics, particularly for continuous, contact-rich tasks like cleaning. While motion primitives can also be learned directly in full joint space, a compact, synergy-based representation provides a shared latent coordinate system that simplifies interpretation, modulation, and cross-task composition. We adopt a data-driven framework for representing and reproducing dexterous manipulation trajectories, using cleaning motions as a test bed. To model these movements, we combine Principal Component Analysis (PCA) with Probabilistic Movement Primitives (ProMPs), leveraging hand synergies. While the PCA and ProMP combination itself is established, our focus in this study, is on the cleaning use case and on the compositional generalization across tasks. PCA, applied in joint space, provides a compact, low-dimensional synergy space for coordinated finger movements, while the ProMPs encode the time-varying structure and variability of trajectories within this space. We first recorded a kinematic dataset of human cleaning motions with 20 degrees of freedom (DOF) haptic exoskeleton gloves across thirteen tasks and learn one ProMP per five selected training tasks in the PCA space. This dataset is then used as a basis to learn cleaning motions using the PCA + ProMPs. We demonstrate the ability of the learned primitives to reconstruct and reproduce kinematic patterns in simulation (Shadow Hand) and successfully deploy them on a physical robotic hand (Aeon Robotics). These results indicate that motion primitives, when grounded in synergy-informed coordinates, can generalize beyond grasping to encode and modulate contact-rich dexterous manipulation skills. Moreover, a library of the five task-specific ProMPs compositionally approximates trajectories from eight unseen cleaning tasks, with nearest-expert selection outperforming convex blends and Product-of-Experts combinations.

## 1 Introduction

Learning movement from data through movement primitives has become a popular approach in robotics (Saveriano et al., 2023a). Movement primitive (MP) frameworks offer compact, reusable representations of trajectories that can be adapted to new conditions with limited computation. Within the broader field of Learning from Demonstration (LfD), MPs have been widely used to encode human-like motions at the whole-body or arm level, for tasks such as reaching, hitting or throwing, and wiping at the end-effector level (Schaal, 2006; Ijspeert et al., 2013). In most of these works, the focus lies on the arm or end-effector trajectory, and the hand is either simplified or treated only at a coarse level (Pignat and Calinon, 2017; Cohen et al., 2021).

With the increasing availability of anthropomorphic robotic hands, many works have studied hand coordination, from grasping to various forms of in-hand manipulation. Most of this literature, however, focuses on relatively simple behaviours, such as stable grasps, short regrasp motions, or finger-gaiting in restricted settings, and often uses simplified hands or heavily constrained motions (Starke and Asfour, 2024; Zhao et al., 2020; Krug and Dimitrov, 2015). In contrast, much less work addresses dexterous manipulation that requires sustained, finger-level coordination during continuous contact with the object, while also providing compositional movement primitives that can be reused and generalized across related tasks within the same category, such as different kinds of cleaning.

From existing work on MPs and synergies, we can identify the following desiderata for a useful representation of dexterous cleaning motions:

- Low-dimensional synergies: capture realistic multi-finger kinematics coordination patterns, rather than treating each joint independently.
- Probabilistic multi-demo modeling: handle multiple demonstrations per task as a distribution, encoding variability and enabling conditioning.
- Robot-ready mapping: provide a simple, well-conditioned inverse mapping from latent space to joint space, without costly optimization at execution time.
- Cross-task generalization: support reuse and composition of a small set of learned MPs to approximate new, related cleaning tasks.

This motivates our central research question: Can we obtain a generalized representation of kinematics manipulation trajectories that captures sophisticated, finger-level movements in contact-rich cleaning tasks and is simple enough to be deployed on real robotic hands? Addressing this question is non-trivial, as contact dynamics and continuous adjustments at the fingertips introduce variability that motivates representations enabling compact models and cross-task composition, while remaining accurate in joint space.

We focus on cleaning motions as a representative test bed. Cleaning is a relevant and practically important application area, with increasing industrial interest and numerous startups targeting domestic, healthcare, and facility-maintenance scenarios (Li et al., 2025; Lew et al., 2022). Crucially, cleaning encompasses a rich set of contact-rich, directionally structured, and repetitive movements at the finger level, making it an ideal domain to evaluate whether MPs can encode dexterous manipulation beyond grasping. Our study is conducted in the broader context of developing robots capable of performing reliable household and service tasks.

Thus, we investigate whether a synergy-based PCA + ProMP representation can serve as such a generalized model of dexterous cleaning motions with the following hypotheses:

- Synergy compression: we ask whether cleaning trajectories can be compressed into a low-dimensional synergy space without losing essential structure. Concretely, we test whether a PCA basis with 5 components for 15 DOFs and 7 components for 20 DOFs can explain at least 90% of the variance in human cleaning motions while keeping the average joint-space reconstruction error below 10%.
- Faithful robot reproduction: we examine whether MPs learned in this synergy space can be faithfully executed on robots. We evaluate whether ProMPs trained in the PCA space generate trajectories that, once mapped back to joint space, remain kinematically close to the demonstrations and can be replayed on both a simulated Shadow Hand and a physical humanoid hand with high success rate in terms of force performance, precision, representativeness, and repeatability.
- Cross-task compositionality: we assess cross-task compositionality. We test whether a small library of task-specific ProMPs learned from five cleaning tasks can be combined to approximate trajectories from eight previously unseen cleaning tasks (via best-single selection and convex blends of expert mean trajectories in the PCA space, and a Product-of-Experts combination in the ProMP weight space) with reasonably low reconstruction errors, indicating meaningful cross-task generalization.

To study this problem, we recorded a comprehensive dataset of human finger-joint kinematics during cleaning tasks, as such data are scarcely available in existing resources. We then adopt a combined PCA with ProMPs: PCA, applied in joint space, provides a low-dimensional synergy space for finger coordination, and ProMPs model distributions over trajectories within this space. Embedding PCA in a ProMP framework yields a probabilistic, time-parameterized generator of finger trajectories that reproduces typical executions and supports modulation via conditioning (e.g., via-points) and temporal scaling.

We evaluate the learned representations of PCA + ProMP in both simulation and on a physical robotic hand. Across cleaning tasks, the PCA + ProMP framework accurately reconstructs and reproduces kinematic patterns of dexterous finger motion, demonstrating robust execution and adaptability under moderate variability. We further show that a small library of five task-specific ProMPs can compositionally approximate trajectories from eight held-out cleaning tasks in the same PCA space. Finally, we map each hypothesis to a dedicated evaluation: *Synergy compression* to PCA variance and reconstruction analysis. *Faithful robot reproduction* to deployment on a 20-DOF simulation and a 15-DOF physical hand with rubric-based scoring. And *Cross-task compositionality* to cross-task reconstruction via best-single selection and convex blends in the shared PCA space, together with a Product-of-Experts combination in the ProMP weight space. These results suggest that motion primitives, when grounded in synergy-informed coordinates, can generalize beyond grasping to contact-rich manipulation.

Our contributions in this paper are as follows:

- We apply a PCA + ProMP approach that couples synergy-based dimensionality reduction with probabilistic trajectory encoding for dexterous cleaning manipulation.
- We validate the deployment of the learned primitives in simulation and on a physical humanoid robotic hand, showing accurate reproduction of cleaning motions and meaningful cross-task generalization.
- We record a dataset of 20-DOF finger-joint kinematics for 13 cleaning tasks.

## 2 Related work

### 2.1 Hand and manipulation datasets

Public hand-object interaction datasets have primarily targeted grasping or pose estimation, rather than fine, finger-level manipulation. Early datasets emphasized contact maps and grasp labels (Brahmbhatt et al., 2019; Saudabayev et al., 2018). Subsequent large-scale datasets expanded to full-body, richly labeled human-object interaction scenes and bimanual interactions (Taheri et al., 2020; Fan et al., 2023; Zhan et al., 2024; Krebs et al., 2021). A few works address cleaning-like activities (Kim et al., 2020), and very recent efforts further scale up hand data (Fu et al., 2025).

Nevertheless, time-aligned, multi-DOFs finger kinematics for contact-rich, continuous tasks, especially cleaning, remain underinvestigated. Our dataset addresses this gap by providing 20-DOF finger-joint trajectories suitable for synergy-based movement-primitive learning.

### 2.2 Principal component analysis for dimensionality reduction

At the level of human motor control, classical studies have shown that grasping is organized through low-dimensional synergies in both hand posture and contact forces. For postural synergies, Santello et al. (1998) demonstrated that static grasp postures across many objects can be explained by a small number of principal components. Mason et al. (2001) reported that reach-to-grasp movements can be described by a small number of coordinated patterns of joint motion, rather than independent finger DOFs. Complementarily, Santello and Soechting (2000) showed that humans also regulate multi-finger contact forces through force synergies during grasping.

Together, these results provide a conceptual basis for synergy models and motivate low-dimensional hand representations, such as the PCA space adopted in our work for learning and controlling dexterous manipulation skills.

In this context, we use PCA for three practical reasons: Firstly, principal components can be directly related to groups of joints or fingers and are easy to retarget across platforms. The second reason is due to the robustness aspect in our data regime (closed-form, well-conditioned estimation with limited overfitting risk). Thirdly, PCA provides a stable, linear, and invertible mapping back to joint space, which simplifies execution of joint limits. PCA is also a widely used baseline for dimensionality reduction in motor-control and biomechanics applications (Daffertshofer et al., 2004; Deluzio and Astephen, 2007) and remains competitive with more complex nonlinear methods in many practical settings. For broader overviews of linear and nonlinear dimensionality reduction, see, for example, Jolliffe (2011), Jolliffe and Cadima (2016), van der Maaten et al. (2009). Prior comparative studies have further reported that linear PCA can achieve performance comparable to nonlinear embeddings on several real-world datasets, suggesting that linear models remain a strong and robust baseline (Patel et al., 2015).

### 2.3 Learning from demonstration with movement primitives

LfD has become a central paradigm for robot skill acquisition. Two canonical MP families dominate the literature: Dynamic Movement Primitives (DMPs) (Schaal, 2006; Ijspeert et al., 2013) which are stable, re-timeable dynamical systems, and Probabilistic Movement Primitives (Paraschos et al., 2013), which provide a Gaussian formulation enabling conditioning, co-activation, and uncertainty handling. In practice, these approaches are often deployed at the arm/end-effector level, either transferring human demonstrations to robots or learning directly on hardware for various tasks, such as point-to-point reaching, throwing, and grasp preshape/closure (Amor et al., 2012; Cohen et al., 2021; Bitzer and Vijayakumar, 2009). In principle, both DMPs and ProMPs can be applied directly in high-dimensional joint spaces. In practice, however, dexterous, finger-level manipulation with anthropomorphic hands involves many coupled DOFs and multiple demonstrations per task, which makes parameter estimation, numerical conditioning, and interpretation less convenient. To mitigate this issue, many works combine dimensionality reduction with MPs. Nonlinear embeddings learn a smooth latent manifold (e.g., a structure-regularized Gaussian Process Latent Variable Model) in which DMPs are encoded and modulated (Bitzer and Vijayakumar, 2009), while other approaches learn a low-dimensional mapping from task parameters to DMP parameters using PCA/LLE (Locally Linear Embedding) and kernel or neural regressors for run-time adaptation (Cohen et al., 2021).

Other methods rely on the extraction of latent variables to capture movement variability and style: for instance (Matsubara et al., 2010), learn “stylistic” DMPs from multiple demonstrations by introducing low-dimensional style variables that parameterize families of movements, and (Rueckert et al., 2015) extract low-dimensional control variables for movement primitives using probabilistic latent variable models to facilitate policy search and adaptation.

On the probabilistic side, dimensionality reduction with the use of ProMPs estimates a shared linear synergy subspace and controls ProMPs therein, improving sample efficiency and policy search (Colomé et al., 2014). For hand manipulation, PCA-based postural synergies provide compact representations that support MPs for generating human-like, adaptable grasps (Amor et al., 2012; Starke and Asfour, 2024).

In this landscape, we adopt a simple, linear strategy: we use a joint-space PCA basis as the synergy representation in which ProMPs are learned. As discussed in the previous subsection, this choice provides an interpretable, and robust low-dimensional space. In the present work, we therefore treat PCA as a baseline for synergy-based dimensionality reduction and focus on demonstrating that even this linear representation is sufficient to learn and generalize contact-rich cleaning motions with ProMPs.

The choice of ProMPs as our movement-primitive representation is motivated by their advantages over other movement-primitive formulations as presented in (Paraschos et al., 2013; Paraschos et al., 2018a). Compared to deterministic MPs such as DMPs, ProMPs model a full distribution over trajectories from multiple demonstrations, capturing time-varying variance and inter-joint correlations while remaining compatible with stochastic optimal control formulations. This probabilistic structure supports conditioning on via-points, principled co-activation and blending of primitives, and robust adaptation to changing task conditions and environments, which is crucial in our setting with many coupled finger DOFs, substantial execution variability, and the need for compositional reuse across related cleaning tasks for generalization (Paraschos et al., 2018b).

Despite this rich body of work on grasping and locomotion, applying synergy MPs to contact-rich, continuous cleaning tasks at the finger level remains underexplored. The subsequent sections detail our methodology for addressing this underexplored area.

## 3 Methods

**FIGURE 1:**
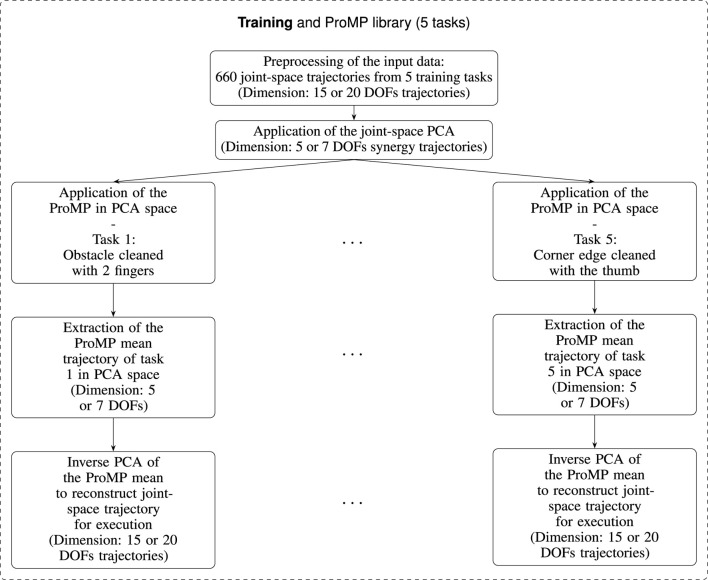
Schematic overview of the PCA + ProMP training pipeline. Joint-space trajectories from five cleaning tasks are projected into a low-dimensional PCA synergy space, and one ProMP is learned per task, yielding a library of five expert primitives whose mean trajectories are mapped back to joint space for execution. For visualization, only tasks 1 and 5 are shown on the graph. The same procedure is applied identically to all five tasks.

In this section, we describe how we construct and evaluate a synergy-based representation of dexterous cleaning motions. We first present the experimental setup used to record human finger kinematics during cleaning. We then detail the selection of cleaning tasks and the recording protocol that yield a diverse dataset of coordinated multi-finger trajectories. Next, we explain how these trajectories are preprocessed and projected into a low-dimensional PCA space, in which we learn one ProMP per task to obtain a compact representation of reusable cleaning skills which will be used on the robotic platform for evaluation. Finally, we describe how these ProMPs are used for cross-task generalization in the synergy space and how the resulting trajectories are mapped back to joint space and executed on the simulated and physical robot hands for quantitative and qualitative evaluation. An overview of the PCA + ProMP *training* pipeline, from joint-space trajectories to task-specific ProMPs and inverse PCA for execution, is shown in Figure 1. This figure summarizes only the training and execution steps. The cross-task generalization procedure is described separately in Section 3.6.

### 3.1 Setup

**FIGURE 2:**
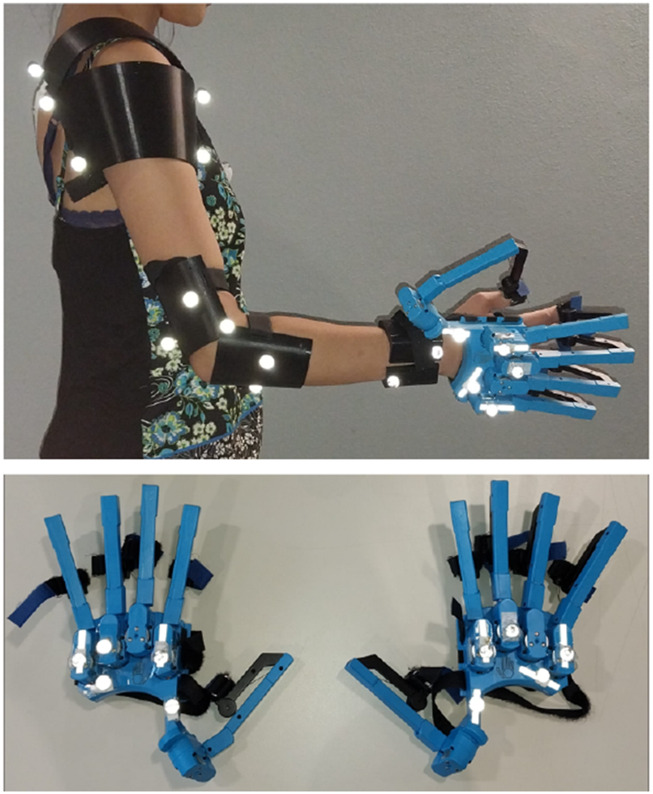
Experimental setup showing the OptiTrack markers on the arm and the SenseGlove DK1 on the hand.

To capture manual kinematics, we recorded full upper-limb and hand kinematics. Arm motion was captured with an OptiTrack motion capture system, and finger-joint kinematics were measured using bilateral haptic exoskeleton gloves (SenseGlove DK1) (Figure 2).

The SenseGlove provides finger-motion tracking with up to 20 joint-angles across the five digits, yielding robust finger-joint measurements. For the index, middle, ring, and little fingers, we recorded MCP, PIP, and DIP flexion/extension. For the thumb, we recorded the CMC, MCP, and IP flexion/extension. Abbreviations are reported in the Table 1. The device exposes both passive kinematic readouts and active brake actuation. In active mode, the glove allows brake control for haptic feedback. In passive (recording) mode, an auxiliary sensor also provides abduction/adduction measurements for each finger. In this study, we used only the passive kinematic channels. As a result, we can record the complete 20-DOF output of the SenseGlove described earlier (covering flexion/extension and abduction/adduction).

**TABLE 1:** Abbreviations of the finger joints.

Abbreviation	Full term
MCP	Metacarpophalangeal joint
PIP	Proximal interphalangeal joint
DIP	Distal interphalangeal joint
CMC	Carpometacarpal joint
IP	Interphalangeal joint

Each participant (for both hands) completed a calibration procedure following the SenseGlove Robot Operating System (ROS) package documentation (SenseGlove, 2025). To calibrate the SenseGloves, participants performed a series of thumb-to-fingertip contacts (thumb to index, middle, ring, and little) to align sensor readings and ensure accurate joint tracking prior to the experimental trials. Calibration and experimental trials were conducted sequentially, one hand at a time. The effective sampling rate of the SenseGlove during data collection was approximately 97Hz.

To evaluate how well the generalized motion model works in practice, we tested it on two robotic platforms: a simulated hand and a physical humanoid robotic hand.

This dual evaluation allows us to verify that the learned representation transfers both in simulation and on real hardware.

We use a simulated robotic hand, the Shadow Hand, which has 24 DOFs, of which 20 will be used in this study. For the four fingers of the Shadow hand, the model includes MCP, PIP, and DIP flexion/extension, and abduction/adduction. For the thumb, CMC, MCP, and IP flexion/extension, as well as thumb abduction/adduction are available. This configuration provides joint-level control over the full dexterous hand for trajectory reproduction.

**FIGURE 3:**
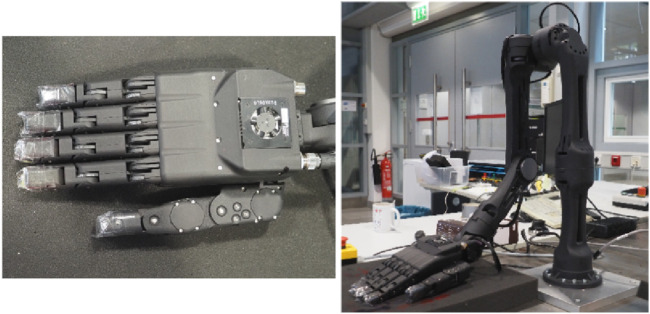
Upper-limb humanoid robot *DROID* from the company Aeon Robotics, and the robotic hand the *Handeffector*.

For real-robot validation, we used a humanoid robotic hand manufactured by the company Aeon Robotics, the *Handeffector* (Figure 3). The device has a total of 15 DOFs, of which 11 are actively controlled. Each of the four fingers comprises 2 active DOFs (MCP and PIP) and 1 passive DOF (DIP), and the thumb provides 3 active DOFs. The hand is instrumented with force-feedback sensors on each of the 11 active DOFs, enabling real-time acquisition of contact force signals during execution.

For the Shadow Hand simulation, we use a calibrated joint-to-joint mapping to retarget the recorded human kinematics to the robot hand. The SenseGlove and the Shadow Hand expose closely matching kinematic structures (for the Shadow Hand: MCP, PIP, and DIP flexion/extension and abduction/adduction for the four fingers, and CMC, MCP, and IP flexion/extension plus thumb abduction/adduction for the thumb), which allows us to define a one-to-one correspondence between glove joints and simulated joints. During an initial calibration sequence, participants perform a set of key poses such as a flat hand, a closed fist, and thumb-to-fingertip contacts for each finger. For each corresponding human/robot joint pair, we estimate an affine mapping (offset and gain) that aligns the glove angle range with the robot joint range and then apply saturation to the robot’s mechanical limits during execution. In the Shadow Hand simulation, the recorded joint trajectories from the SenseGlove are therefore applied directly to the corresponding Shadow Hand joints through this calibrated mapping. This joint-to-joint retargeting strategy is in line with embodiment mapping approaches proposed for anthropomorphic hands (Gioioso et al., 2013; Liarokapis et al., 2013; Li et al., 2021).

For the physical Aeon hand, we rely on the manufacturer’s SenseGlove-based teleoperation interface, which implements a Cartesian mapping from human fingertip poses to robot finger joint commands. In this interface, the glove provides fingertip positions and joint angles, and a kinematic model of the human hand together with per-finger inverse kinematics is used to compute the desired joint commands for the robotic fingers. The mapping on the Aeon platform is therefore defined at the level of fingertip pose rather than as a pure encoder-to-encoder calibration, but it preserves a consistent one-finger-to-one-finger correspondence and qualitatively similar motions between operator and robot.

All learning and ProMP modeling are conducted in joint space. Cartesian mappings are only used in the final retargeting to the physical hand.

### 3.2 Selection of experimental tasks

The thirteen cleaning tasks were not chosen arbitrarily. Before designing the protocol, we informally observed how people clean everyday surfaces (tables, corners, objects on a support) and noted common patterns in hand posture, contact regions, and environmental obstacles. We then selected a set of objects (e.g., bowl, curved bottle, sunglasses) and surface configurations (flat areas, edges, corners, obstacles) that naturally induce different multi-finger coordination strategies. As a result, the thirteen tasks capture different cleaning situations while remaining structured enough for repeatable data collection and analysis.

For this study, we focused on five cleaning tasks out of the thirteen we recorded (Table 2). Our selection followed the *Virtual Fingers* (VFs) framework introduced by Iberall (1986) and later used in Feix et al. (2015), which conceptualizes the hand as organizing fingers into functional units to accomplish actions. Within this framework, task complexity and coordination can be characterized by the number and configuration of these units. We expanded on the concept of VF by distinguishing between active and passive finger groups for each task, adding an additional layer to our analysis. Guided by this prior work, we initially compiled thirteen tasks that demand distinct, well-coordinated finger motions (for instance, treating the thumb as one VF and the index and middle pair as another VF) so that the resulting dataset would contain rich, complex kinematics suitable for subsequent PCA.

**TABLE 2:** List of the thirteen recorded tasks. In bold, the five tasks used to train the algorithm.

Name of the task	Description
Bowl	Wiping the bowl’s rim with the index, middle, ring and little fingers
Curved bottle	Polishing up to down along a curved bottle with all fingers
**Sunglasses**	**Cleaning sunglasses using the thumb with the index and middle fingers**
Dusting	Sweeping dust from a tabletop with the full hand
Edge of a table	Wiping the table edge (non-corner) with the index to little fingers
**Obstacle cleaned with 2 fingers**	**Wiping along an obstacle’s edge with the index and middle fingers without crossing above it**
Obstacle cleaned with 1 finger	Wiping along an obstacle’s edge with the index finger without crossing above it
**Obstacle cleaned with the thumb**	**Wiping along the same obstacle with the thumb**
Obstacle cleaned with the full hand	Wiping along the same obstacle with the entire hand
**Corner of a table cleaned with the thumb (rotational)**	**Scrubbing a table corner with a rotational thumb motion**
**Corner edge cleaned with the thumb**	**Wiping along the corner edge with the thumb**
Corner of a table cleaned with 4 fingers (rotational)	Scrubbing a table corner with a rotational full-hand motion
Corner of a table cleaned with 2 fingers (rotational)	Scrubbing a table corner with a rotational motion of the index and middle fingers

**FIGURE 4:**
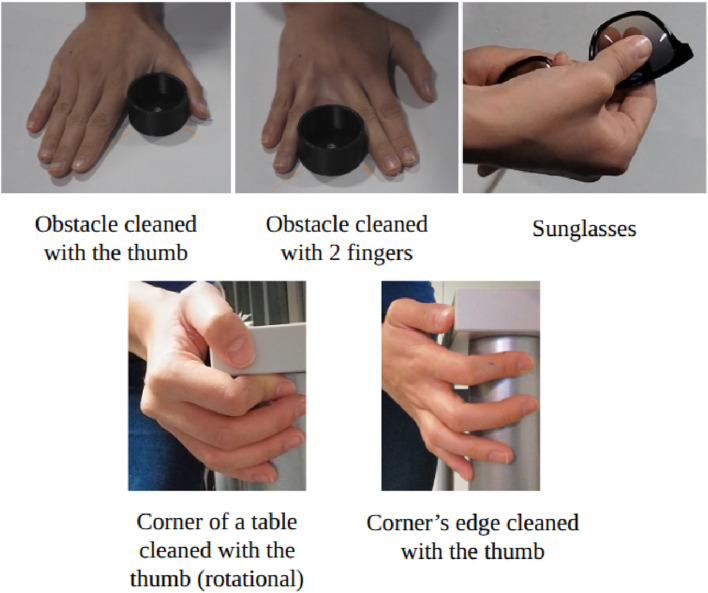
The five selected movements, which will be used as a training set for the algorithm.

From those thirteen, we selected five tasks that displayed the widest range of VF configurations (Figure 4): (1) scrubbing a table corner using a rotational motion of the thumb, (2) wiping along the corner edge with the thumb, (3) cleaning along an obstacle’s edge with the index and middle fingers while avoiding motion above the obstacle, (4) cleaning the same obstacle with the thumb, and (5) cleaning sunglasses using the thumb together with the index and middle fingers. In the remainder of this paper, we refer to the task as labeled in Figure 4.

The “obstacle cleaned with 2 fingers” task was chosen in this context to isolate a simple case where only two fingers are constrained. In this task, the index and middle fingers must follow the edge of the obstacle without crossing above it, while the other fingers remain free. In the VF framework, this allows us to isolate an index and middle VF under some geometric constraints and to study how the rest of the hand passively moves when only this pair is actively constrained. Together with the other tasks, where we constrain only the index, only the thumb, or the full hand, this helps us analyse how active virtual fingers influence the motion of non-constrained fingers.

This focused subset concentrates our analysis on the most complex and representative movements in the dataset.

The decision to focus on only five tasks, rather than the full set of thirteen, was driven by a key objective. This approach enables us to test one of the core functionalities offered by the ProMP framework. The compositional generalization of skills (Paraschos et al., 2013) that will be explained in Section 3.6. By training a limited library of distinct prototypes (the five selected tasks), we can later evaluate whether the trajectories from the eight unseen tasks can be accurately reconstructed as a composition of the learned primitives. This constitutes a crucial test of our framework’s representational power and its capacity to extend learned skills to novel motions.

### 3.3 Recording of data

A cohort of 22 healthy adults participated (10 left-handed, 12 right-handed). The sample included 5 women and 17 men, aged 23–61 years, with a mean age of 30.26 ± 9.28 years. All participants provided written informed consent prior to enrollment.

**FIGURE 5:**
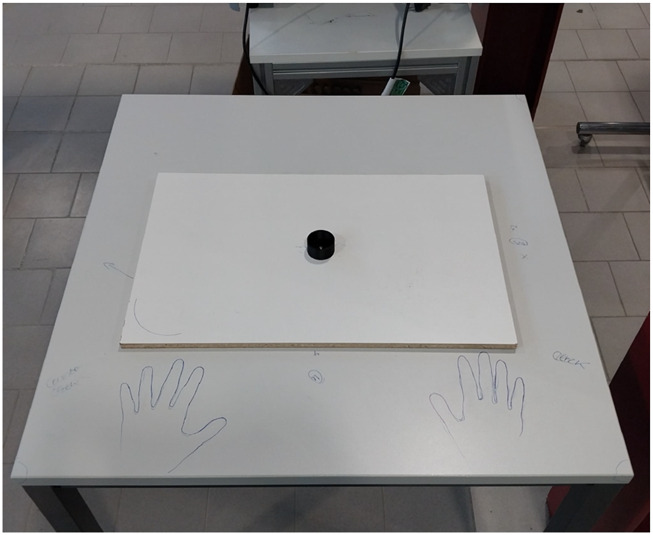
Experimental table for the data acquisition.

Each participant carried out the full set of thirteen cleaning tasks, with three recordings per task and five repetitions per recording to be performed by the dominant and non-dominant hand, meaning each hand, for all the participants had to perform 13 tasks one after the other. As described in the previous subsection, only five tasks were retained for the present analysis. This yielded 330 cleaning recordings per hand, calculated as 5 tasks × 22 participants × 3 recordings for one hand. This totals 660 recordings across both hands for all the participants (for the dominant and non-dominant hand) over a total of 1716 recordings for all the tasks and participants. Participants were instructed to execute the movements using the active DOFs of the hand, as defined earlier. Passive DOFs were not constrained. Some finger positions were partially restricted by the apparatus (e.g., maintaining contact with a pole or the tabletop in specific tasks). To standardize execution, a demonstration video of each task was shown. Supplementary Videos illustrating the movements are available. Task order and the choice of which hand was used first were randomized across participants. For each recording, the procedure was as follows: the participant placed the hand flat on the experimental table at a marked starting position (Figure 5). Upon the start cue, people moved their hand to the target surface, completed five movements, and ended the recording by returning to the initial position with the hand flat on the table. The dataset can be obtained from the authors upon request.

### 3.4 Preprocessing and application of PCA

In this section, our objective is to extract low-dimensional joint-synergies that compactly describe coordinated multi-finger kinematics and provide a convenient latent space for subsequent joint-space trajectory learning.

To this end, we preprocess the data separately for each participant and task prior to PCA. We retain only the time windows during which the fingers are in contact with the target surface. From these windows, we assemble a matrix of segmented trajectories that serves as the PCA input. Following established practice (Cushion et al., 2019; Daffertshofer et al., 2004; Deluzio and Astephen, 2007), we apply the PCA on the joint-space to obtain a compact representation of hand kinematics. PCA maps the original variables, here the hand’s DOFs, to an orthogonal set of principal components ordered by explained variance, thereby reducing dimensionality while preserving dominant covariation patterns (i.e., candidate synergies).

Each recording, regardless of the task, contains five repetitions. We segment these into five parts (hereafter referred to as *segments*). We remove the initial and final phases in which the hands rest on the surface to isolate the cleaning motion. To avoid transient effects and atypical trajectories, we additionally discard the first and last repetitions, allowing participants to stabilize their execution and eliminating end-of-recording lift-off artifacts. The remaining segments are smoothed with a Gaussian filter and denoised with a median filter to suppress local spikes.

Applying PCA directly to raw time series is confounded by variable segment durations. Because PCA requires a fixed feature length per observation, we time-normalize all segments to a uniform length via cubic interpolation. The normalized segments are then stacked into a single matrix containing all the tasks. Let d denote the number of DOFs and T the normalized segment length (in samples). We construct a data matrix:

$$X\in R^{\left(T\times R\right)\times d}$$

where R is the number of retained segments (after exclusion). Columns of the matrix correspond to DOFs. Rows correspond to time samples across segments and multiple recordings of all tasks and participants, concatenated for a pooled analysis. Meaning the rows correspond to: the time of a performed trajectory × sample rate (same for all the trajectories whatever the task, hand, or participants) × number of tasks × 3 recordings per task × number of participants × 2 hands. This organization is required to identify the principal modes of covariation across DOFs. PCA is performed on the standardized (z-scored) matrix X.

We report the explained variance and retain the leading components for trajectory reduction, which are subsequently used in the learning stage (e.g., training motion models on low-dimensional coefficients).

Because we evaluate our method on two robots with different number of DOFs, and because PCA depends on the input variables, we split the study into two tracks. For the physical robot, which focuses on flexion/extension motions (DIP, PIP, MCP for the fingers. CMC, MCP, IP for the thumb), we build a PCA input matrix using only those DOFs, which leads to 15 DOFs. In parallel, to stay faithful to a human hand, we construct a 20-DOF matrix (flexion/extension plus adduction/abduction) and use it on the simulated robotic hand, the Shadow Hand. This separation maximizes the explained variance for each platform by focusing on the DOFs each robot can actually realize.

### 3.5 Application of the ProMPs in the PCA space

To learn cleaning joint-trajectories and obtain a generalized model per task, we employ the ProMPs in the low-dimensional PCA space, following the framework introduced by (Starke and Asfour, 2024). A ProMP models a family of time-normalized trajectories as a Gaussian distribution over basis-function weights rather than a single deterministic curve. Concretely, for a trajectory sample at phase/time t , we have the following Equation 1:

$$y_{t}=\Phi_{t}^{⊤}\;w+\varepsilon_{t},\;\text{with}\;w∼N\left(\mu_{w},\Sigma_{w}\right),\;\varepsilon_{t}∼N\left(0,R\right),$$ (1)

where $\Phi_{t}$ stacks M temporal basis functions (e.g., Gaussians) and $y_{t}$ denotes the k-dimensional coordinates of the hand posture at time *t* expressed in the PCA basis (i.e., the PC scores). Learning joint-trajectories from multiple demonstrations estimates the weight distribution $N\left(\mu_{w},\Sigma_{w}\right)$ , which captures average motion and its natural variability/covariation across DOFs.

In our framework, we first partition the preprocessed data by task. In our case, we work with five tasks, leading to five corresponding ProMPs.

As described in the PCA subsection and in Figure 1, trajectories were filtered, time-normalized, centered, spatially normalized, and segmented. Each recording contains five repetitions, after discarding the first and last repetitions to avoid transients and lift-off artifacts, each recording contributes three valid segments. Per task, this yields 22×3×2=132 trajectories (participants × retained segments per recording × hands). For each task, ProMPs are trained in the corresponding PCA space, which is derived from either the 20-DOF or the 15-DOF joint-space representation, each yielding a low-dimensional set of principal components. The resulting PCA coefficient trajectories are used directly as observations $y_{t}$ for ProMP training.

We then fit one ProMP per task on the coefficient trajectories. We use M=20 Gaussian basis functions per DOF in all experiments (centers evenly spaced over phase, fixed width). This choice is supported by the ablation study reported in Section 4, where we vary M (number of basis functions) and show that reconstruction errors saturate around M=15 -20.

Parameter estimation follows the standard closed-form procedure: for each demonstration, ridge regression yields w from $\left\{y_{t},\Phi_{t}\right\}$ , and $\left(\mu_{w},\Sigma_{w}\right)$ are obtained as the sample mean and covariance over all demonstrations (equivalently, a Gaussian MLE).

In all experiments, we use a normalized phase variable t∈[0,1] and a ridge-regularization parameter $\lambda =10^{-6}$ when estimating the individual weight vectors.

For execution, we extract the generated mean trajectories of the ProMP and map them back to joint space by inverse PCA, followed by platform-specific retargeting to joint limits. The learned ProMPs can be adapted to task constraints (e.g., targets, via-points, start or end states). They also keep the natural coordination between the PCA components through the covariance $\Sigma_{w}$ , and they reproduce movements on the robot while taking into account the variability observed in the demonstrations.

These five task-specific ProMPs form a small set of reusable cleaning skills. We employ them for cross-task generalization in the PCA space, and we also derive temporally and spatially modified variants by acting on their mean and variance (e.g., via time scaling and via-point conditioning). The resulting trajectories are then replayed on both the simulated Shadow Hand and the physical humanoid hand to assess whether the robot effectively reproduces the intended cleaning motions.

### 3.6 Cross-task generalization with ProMPs (product-of-experts and convex blends)

**FIGURE 6:**
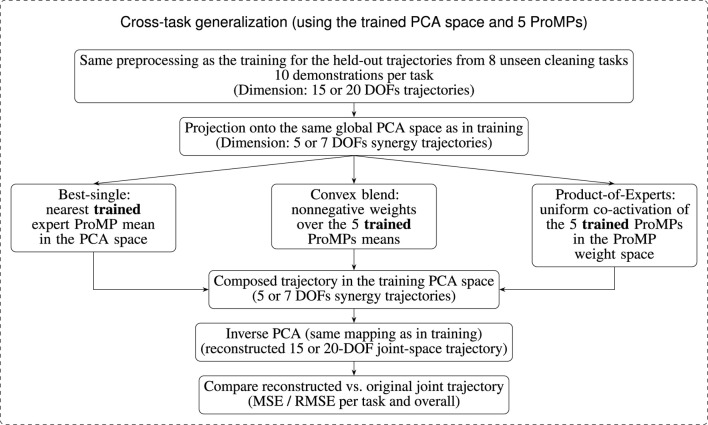
Schematic overview of the cross-task generalization procedure. Trajectories from eight unseen cleaning tasks are preprocessed and projected onto the same global PCA synergy space that was learned during training. They are then approximated using the same library of five task-specific ProMPs (best-single expert, convex blend of ProMP means, or Product-of-Experts). The composed trajectory in this PCA space is mapped back to joint space via the same inverse PCA and compared to the original demonstration using the mean squared error (MSE) and the root mean squared error (RMSE).

We learn five task-specific ProMPs in the global PCA space, one per selected cleaning task (see Section 3.5). These ProMPs form a small library of expert skills, each represented as a Gaussian distribution over trajectories in the low-dimensional synergy space (Paraschos et al., 2013; 2018a). To assess cross-task generalization, we use this library to reconstruct trajectories from the eight held-out (unseen) cleaning tasks as follows. The overall cross-task generalization pipeline, including projection, composition, and reconstruction, is summarized in Figure 6.

For each unseen task, we take ten held-out demonstrations and process them exactly as in the training pipeline (segmentation, time-normalization, filtering). Each resulting joint-space trajectory is projected onto the global PCA basis, yielding a time-varying coefficient trajectory (a curve of 5 or 7 synergy coefficients, depending on the DOF configuration). Given such a target trajectory in PCA space, we construct a composed trajectory using three composition mechanisms which will help reconstructing the held-out trajectories:

- Best-single selection: We identify the expert ProMP whose mean trajectory in PCA space is closest to the held-out coefficient curve in a least-squares sense, and we use this mean curve directly as the composed trajectory. This provides a simple, interpretable baseline that answers whether an unseen movement is essentially covered by one learned prototype (inspired by Gao et al. (2024)).
- Convex blend over expert means: Instead of committing to a single expert, we form a weighted sum of the five expert mean trajectories (Paraschos et al., 2013; 2018a). The nonnegative mixing weights, constrained to sum to one, are obtained by ridge-regularized least squares so that the blended mean trajectory best approximates the held-out coefficient curve. This convex blend tests whether the unseen movement lies in the linear span of the learned skills and yields interpretable coefficients (e.g., sparse versus diffuse use of experts).
- Product-of-Experts (PoE): Following Paraschos et al. (2013), we combine the Gaussian distributions over ProMP weights by multiplying the five expert weight-space Gaussians with uniform activations. This yields a single new ProMP whose weight mean and covariance tighten where experts agree. Its mean trajectory, expressed in the same PCA synergy space as the other experts, is then used as a target-independent co-activation baseline and compared to all held-out trajectories.

For each held-out demonstration and each composition mechanism, the resulting composed trajectory in synergy space (5 or 7 dimensions) is mapped back to the full 15- or 20-DOF joint space via the inverse PCA transform, yielding a reconstructed joint trajectory for the hand. Cross-task generalization is then quantified by comparing these reconstructed joint-space trajectories against the original held-out demonstrations using the Mean Squared Error (MSE) and the Root Mean Squared Error (RMSE).

## 4 Results

### 4.1 Application of the PCA for dimensionality reduction

To reduce the dimensionality of the trajectories to be learned, we first apply PCA so that subsequent modeling operates in a low-dimensional subspace N<20 (the original joint space has 20 DOFs). PCA is a statistical technique for dimensionality reduction that transforms correlated variables into a new set of Principal Components (PCs), ordered by their individual explained variance. In our context, we are interested in the cumulative explained variance (the proportion of total variance accounted for by the first k PCs) together with the PCs themselves, in order to determine an appropriate low-dimensional representation.

We run PCA on two global input matrices aggregating all recordings of the five selected tasks regardless of participant, or hand: one matrix for flexion/extension DOFs (15-DOF configuration) and one for abduction/adduction (20-DOF configuration).

Prior to PCA, trajectories are already preprocessed and time-normalized following the method described in the Section 3.4. They are also spatially normalized and z-scored (centering to zero mean with unit variance per DOF).

**FIGURE 7:**
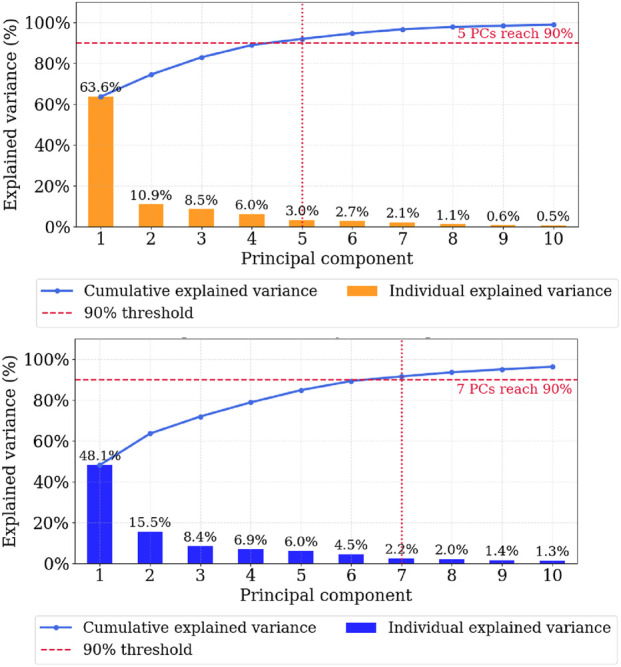
Cumulative explained variance for the cleaning dataset. (Top) Variance explained for the 15 DOFs subset (flexion/extension only). (Bottom) Variance explained for the full 20 DOFs set (including abduction/adduction). The number of principal components k required to reach 90% explained variance is indicated by the red dotted lines in both plots.

We then determine the minimum number of PCs required to reach a target cumulative explained variance of 90%, a threshold chosen to preserve most of the kinematic variability while enabling compact modeling. Under this criterion, the cumulative explained variance curves are reported in Figure 7. From these plots, we retain 5 PCs for the flexion/extension configuration and 7 PCs for the abduction/adduction configuration. For comparison, the commonly used thresholds of 80% and 95% yield 3/7 PCs in the 15-DOF case and 5/9 PCs in the 20-DOF case, respectively.

In practical terms, this means that, rather than learning trajectories in the full 20-DOF joint space, projecting them into the PCA space reduces each trajectory to a k-dimensional coefficient sequence (i.e., k = 7 in the abduction/adduction case, and k = 5 for the 15-DOF case), which is then used for subsequent learning.

**FIGURE 8:**
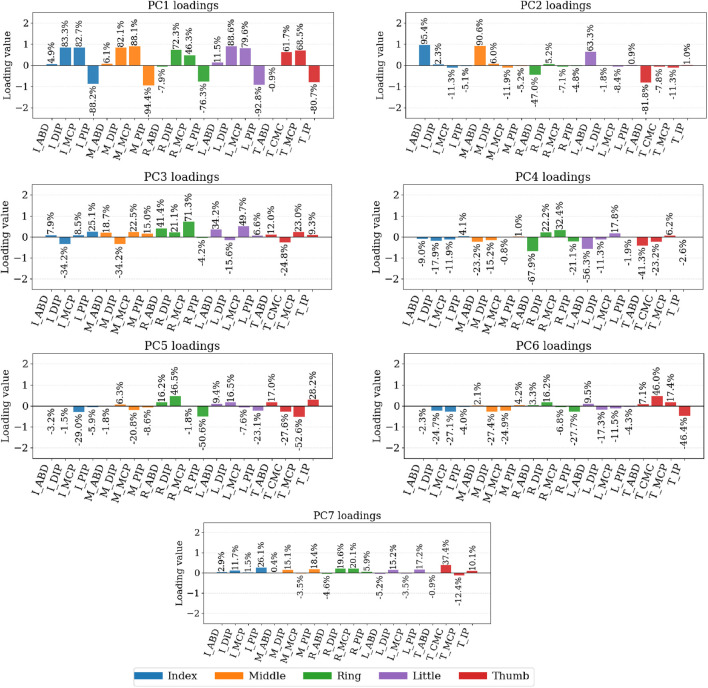
Principal component loadings computed from the cleaning dataset. Spatial loadings of the principal components for the full 20-DOF kinematic model. We display the 7 PCs which express 90% of the variance. The capital letter “I″ stands for “index” finger. Similarly, “M″ stands for “middle finger”, “R”: ring finger, “L”: little finger, “T″ for the thumb.

**FIGURE 9:**
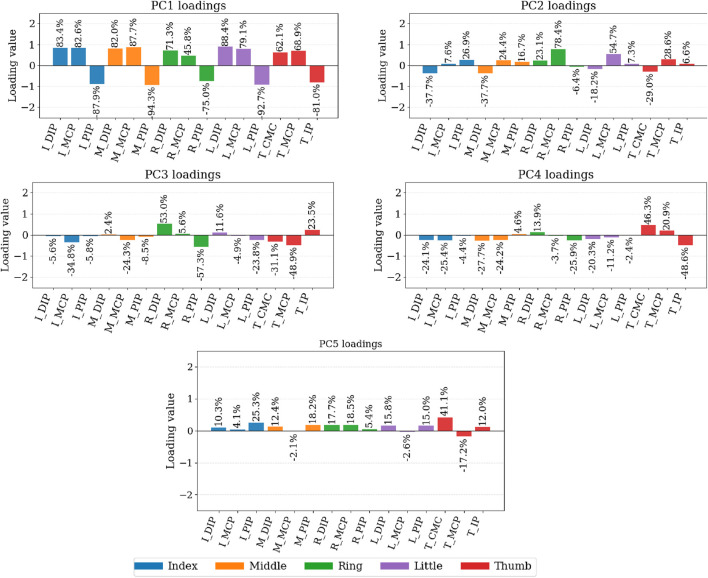
Principal component loadings computed from the cleaning dataset. Spatial loadings of the principal components for the reduced 15-DOF subset (flexion/extension only). We display the PCs which express 90% of the variance. In the case of 15 DOFs, we have 5 PCs. Similarly as before, the capital letter “I″ stands for “index” finger. Similarly, “M″ stands for “middle finger”, “R”: ring finger, “L”: little finger, “T” for the thumb.

In addition to variance accounting, we examined the PC loadings (feature–component coefficients) to interpret what each component captures kinematically (Figures 8, 9). The loadings indicate how strongly each DOF contributes to a given component. In the 20-DOF setting, the first principal component (PC1), concentrates near-uniform weights on long-finger flexion (MCP/PIP/DIP), PC2 captures abduction/adduction (hand spread vs. thumb opposition), and higher PCs separate ulnar-side motion (ring/little) and thumb-specific patterns (CMC/MCP/IP). In the 15-DOF setting (flexion/extension only), PC1 reflects a global flexion mode, followed by lateralized refinements (ring/little vs. index/middle) and a thumb-focused component.

**FIGURE 10:**
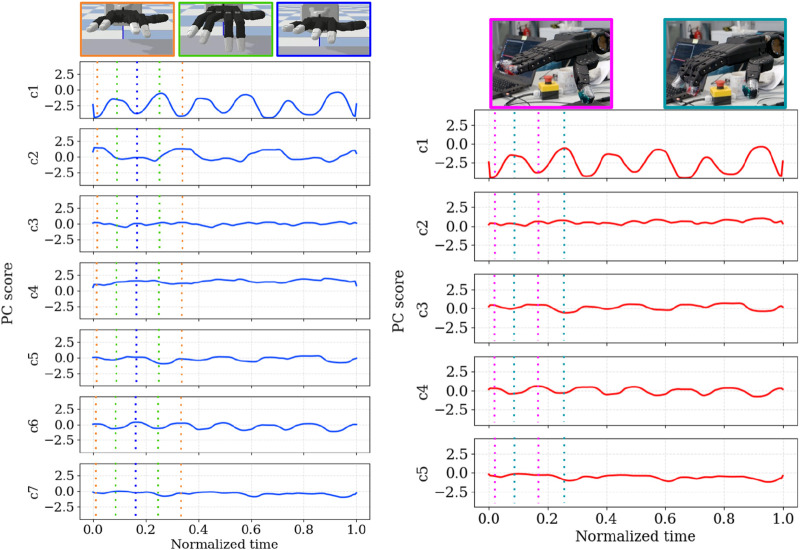
Example time courses of PCA scores for the task *obstacle to be cleaned with 2 fingers* (left: 20-DOF, k=7 ; right: 15-DOF, k=5 ). For each configuration, the score trajectories $c_{i}\left(t\right)$ of the first principal components (PC1, PC2, …  ) are plotted as a function of normalized phase, illustrating how the spatial synergies are activated over time. Each principal component ${\text{PC}}_{i}$ is associated with a score trajectory $c_{i}\left(t\right)$ . Colored vertical dashed lines mark selected time steps t, with corresponding hand postures shown as snapshots framed in matching colors. The curves are slightly smoothed with a moving-average filter (window W=15 samples) for visualization only.

To make the extracted synergies more concrete, Figure 10 illustrates, for a single representative cleaning trajectory, the evolution of the *PCA scores* over normalized time. For this example, we project a time-normalized joint trajectory onto the global PCA basis and plot, for each of the first k components ( k=7 for the 20-DOF setting and k=5 for the 15-DOF setting), the corresponding coefficient $c_{i}\left(t\right)$ as a function of the movement phase t∈[0,1] , with ${\text{PC}}_{i}$ associated to the score trajectory $c_{i}\left(t\right)$ . Consequently, each curve reflects how strongly a given spatial synergy (principal component) is activated over the course of one execution, while the PCA itself remains a purely spatial decomposition over joint coordinates. Consistent with the spatial loadings in Figures 8, 9, the first component PC1, which explains most of the variance, shows the largest excursion in its score $c_{1}\left(t\right)$ and broadly follows the main cleaning phase. Higher-order components contribute smaller score modulations that fine-tune the motion (for example, thumb posture or ulnar–radial refinements).

### 4.2 Reconstruction error and comparison with ProMP-baseline

Once the five cleaning tasks are learned with the PCA + ProMP method, we evaluate the quality of the learned trajectories. As an objective metric, we use the MSE, a standard measure often used in the literature that quantifies the discrepancy between predicted and observed values (Calinon, 2013; Saveriano et al., 2023b; Pignat and Calinon, 2017). We also report the residual standard deviation which quantifies the magnitude of the reconstruction between a demonstration and a reference trajectory. For comparison, we report three MSE measures: PCA + ProMP, ProMP-only, and the empirical mean baseline.

We start with the PCA + ProMP reconstruction errors. For all reconstruction results, each task is represented by a single ProMP in the PCA space (5 or 7 PCs) with 20 Gaussian basis functions per dimension and a normalized phase variable t∈[0,1] . We then generate the mean trajectory of the task-level ProMP on the same phase grid, map it back to joint space by inverse PCA, and compute the MSE and the standard deviation (STD) with respect to each demonstration using all DOFs. In the context of the MSE study, we use the task-level ProMP in its canonical form, without additional tuning, so the error quantifies how well the canonical ProMP for a task represents the whole set of demonstrations. Concretely, for each task we generate the mean trajectory from the corresponding ProMP and compare it against the recorded demonstrations, computing the MSE to assess fidelity (i.e., how closely the learned trajectory matches the real ones).

Per-task PCA + ProMP MSEs for both configurations are reported in Table 3 (a). For the 20-DOF setting, the average joint-space MSE across all tasks is 9.02%. For the 15-DOF setting, it is 8.94%. In both cases, tasks such as cleaning the table corner’s edge with the thumb, cleaning an obstacle with the thumb, and sunglasses contribute slightly more to the overall average because they exhibit higher errors. This higher error can be attributed to greater variability in unconstrained DOFs and finger-placement strategies across demonstrations (e.g., multiple plausible contact configurations). As a result, the ProMP-mean trajectory inherently averages over these alternatives, which leads to slightly higher errors for tasks with more diverse execution patterns. The corresponding residual dispersion is 0.275 (20 DOFs) and 0.268 (15 DOFs), i.e., a residual spread of the same order as the intrinsic inter-demonstration variability.

**TABLE 3:** Per-task joint-space MSE for the five training tasks in the 20-DOF and 15-DOF settings, together with empirical and model-based residual variability.

Task	20 DOFs	15 DOFs
(a) PCA + ProMP reconstruction ( ${\text{MSE}}_{\text{PCA}+\text{ProMP}}\;\pm$ ${\text{STD}}_{\text{resid}}$ )		
Obstacle with thumb	0.09495 ± 0.28057	0.09232 ± 0.26650
Obstacle with 2 fingers	0.07687 ± 0.25813	0.07339 ± 0.24946
Corner edge – thumb	0.09540 ± 0.28719	0.09887 ± 0.28636
Corner rotational – thumb	0.08927 ± 0.27456	0.08530 ± 0.26534
Sunglasses	0.09431 ± 0.27645	0.09721 ± 0.27087
Overall average	0.09016 ± 0.27538	0.08942 ± 0.26771
(b) ProMP-only reconstruction ( ${\text{MSE}}_{\text{ProMP}}\;\pm$ ${\text{STD}}_{\text{resid}}$ )		
Obstacle with thumb	0.09245 ± 0.27531	0.08877 ± 0.25831
Obstacle with 2 fingers	0.07149 ± 0.24733	0.06963 ± 0.23957
Corner edge – thumb	0.09092 ± 0.28022	0.09676 ± 0.28264
Corner rotational – thumb	0.08345 ± 0.26490	0.08269 ± 0.26005
Sunglasses	0.09073 ± 0.27042	0.09476 ± 0.26563
Overall average	**0.08581** ± **0.26764**	**0.08652** ± **0.26124**
(c) Empirical variability of demonstrations ( ${\text{MSE}}_{\text{emp}}\;\pm$ ${\text{STD}}_{\text{emp}}$ )		
Obstacle with thumb	0.09233 ± 0.27500	0.08861 ± 0.25788
Obstacle with 2 fingers	0.07117 ± 0.24660	0.06921 ± 0.23854
Corner edge – thumb	0.09085 ± 0.28009	0.09667 ± 0.28248
Corner rotational – thumb	0.08344 ± 0.26488	0.08268 ± 0.26003
Sunglasses	0.09072 ± 0.27042	0.09476 ± 0.26562
Overall average	0.08570 ± 0.26740	0.08639 ± 0.26091

In Tables (a)–(c), each cell reports the mean MSE (averaged over demonstrations) and a global dispersion measure shown after the ± sign. ${STD}_{\text{resid}}$ is computed from the reconstruction residuals, i.e., the per-demo residual standard deviation averaged over demonstrations. ${STD}_{\text{emp}}$ is computed analogously using the empirical mean trajectory $\bar{y}\left(t,d\right)$ . All STD values are computed over residual samples pooled across time and DOFs (over (t,d) ), per demonstration and then averaged over demonstrations. They are not computed as a standard deviation of MSE scores. For the MSE-type quantities, lower values indicate better reconstruction. Boldface indicates the lowest overall-average joint-space MSE (best reconstruction) among the model-based reconstruction methods (PCA+ProMP vs. ProMP-only).

For comparison, we also trained a ProMP-only baseline applied on each of the five tasks directly in the original joint space, using exactly the same demonstrations, number of basis functions, and training procedure, but without the PCA step.

Table 3 (b) report the resulting joint-space MSEs. Across all five tasks and for both DOF configurations, ProMP-only yields slightly lower reconstruction MSE than PCA + ProMP in our current setting. Between the two methods (PCA + ProMP and ProMP), on average, the MSE decreases from 9.02% to 8.58% in the 20-DOF case, and from 8.94% to 8.65% in the 15-DOF case.

These results indicate that, with our current amount of training data, learning a ProMP directly in joint space does not show a clear reconstruction disadvantage. In terms of STD, ProMP-only shows slightly lower residual dispersion (0.268/0.261 for 20/15 DOFs) than PCA + ProMP, suggesting a marginally tighter fit around the reconstructed mean trajectory.

To relate these reconstruction errors to the variability of the original demonstrations, we additionally compute, for each task and hand, an empirical mean trajectory defined as the average of all demonstrations at each time step and DOF, and report the resulting quantities in Table 3 (c). For every individual demonstration, we then to this empirical mean over all time steps and DOFs. ${\text{MSE}}_{\text{emp}}$ is the average of this quantity across demonstrations, while ${\text{STD}}_{\text{emp}}$ is the corresponding global standard deviation (STD) of these differences.

Across the five training tasks, the empirical MSE is 8.57% (20 DOFs) and 8.63% (15 DOFs). ProMP-only closely matches this empirical baseline (8.58% and 8.65%, respectively), whereas PCA + ProMP yields slightly higher MSE values (9.02% and 8.94%). This reflects the bias introduced by projecting trajectories onto a reduced synergy space (5 or 7 PCs), which trades a small loss in best-fit reconstruction for a compact, shared representation used for composition and control. Moreover, Table 3 compares the empirical dispersion ( ${\text{STD}}_{\text{emp}}$ ) with the residual dispersion of reconstruction errors ( ${\text{STD}}_{\text{resid}}$ ) produced by the ProMPs. The empirical standard deviation exhibits a global standard deviation of approximately 0.26–0.27. PCA + ProMP yields higher average ${\text{STD}}_{\text{resid}}$ (0.275 and 0.268 for 20/15 DOFs) than ProMP-only (0.268 and 0.261), indicating a less concentrated distribution. Across methods, STD values are close to the empirical baseline ( ${\text{STD}}_{\text{emp}}\approx 0.26$ –0.27), suggesting that reconstruction residuals remain within the natural dispersion of the demonstrations.

Moreover, to ensure that the obtained results is not an artefact of a particular choice of the number of basis functions, we conducted an ablation study on the ProMP parameterization. We varied the number of Gaussian basis functions of the ProMP, M∈{2,3,4,5,10,15,20,25,30,35,40} , and we evaluated how this affects reconstruction performance for both the ProMP-only baseline and the PCA + ProMP model.

For each value of M , we trained on the same five cleaning tasks and computed the joint-space reconstruction MSE for all demonstrations in both the 15-DOF and 20-DOF configurations (Table 4).

**TABLE 4:** Average joint-space MSE over the five training tasks as a function of the number of Gaussian basis functions M for PCA + ProMP and ProMP-only, in the 20-DOF and 15-DOF settings. We highlight M = 20, the number of Gaussian basis functions used in our study, as it lies in the plateau region where additional basis functions do not significantly improve the reconstruction error.

Methods	PCA + ProMP		ProMP only	
*M*	MSE (20 DOFs)	MSE (20 DOFs)	MSE (20 DOFs)	MSE (15 DOFs)
2	0.118728	0.123775	0.117271	0.121026
3	0.118691	0.123745	0.117244	0.120997
4	0.111926	0.115143	0.108493	0.115558
5	0.099869	0.102317	0.097040	0.100802
10	0.093849	0.094319	0.089852	0.091836
15	0.090782	0.090245	0.086495	0.087421
20	**0.090161**	**0.089417**	**0.085809**	**0.086522**
25	0.090100	0.089333	0.085731	0.086422
30	0.090086	0.089315	0.085715	0.086402
35	0.090082	0.089310	0.085709	0.086394
40	0.090080	0.089307	0.085707	0.086391

Boldface highlights the main setting/results used throughout the study (M = 20 Gaussian basis functions) because the reconstruction MSE reaches a plateau for M ≈ 15–20.

Averaging over tasks and hands, increasing M reduces the reconstruction error for both methods up to a plateau around M=15 –20 (Table 4). Across the full range of tested M , ProMP-only yields consistently lower MSE than PCA + ProMP in both the 20-DOF and 15-DOF settings. Given the plateau, we fix the number of gaussians to M=20 in all experiments as a consistent setting in the saturation regime.

In relative terms, the reduction in MSE when increasing the number of basis functions from M=5 to M=15 corresponds to roughly 10– 16% of the empirical demo variance reported in Table 3, indicating that these changes are also of practical rather than merely numerical relevance.

Moreover, we benchmark the computational cost of both models on the same machine for M=20 . In the 15-DOF configuration, the ProMP-only pipeline required 102.4 s in total, whereas PCA + ProMP completed the same operations in 13.1 s. For 20 DOFs, the speed-up was even more pronounced (198.8 s for ProMP-only vs. 19.9 s for PCA + ProMP). Peak memory consumption also share the same pattern: for 20 DOF, we obtain 194.84 MB for the ProMP model and 50.07 MB for PCA + ProMP. For 15 DOF, we have 109.66 MB for the ProMP model and 37.41 MB for PCA + ProMP. This differences can be explain by the dimensions of the data. All experiments were run on a computer equipped with an Intel Core i7-6700HQ CPU (4 cores, 8 threads, 2.60 GHz), 16 GB of RAM, and an NVIDIA GeForce 940MX GPU. Overall, these results indicate that, in our setting, operating in a low-dimensional synergy space improves computational cost.

### 4.3 Experiment on the simulated robot hand

To assess whether the learned trajectories are practically deployable and still realize meaningful cleaning motions at the finger level, we first evaluate them on robotic platforms in both the 20-DOF and 15-DOF settings. This stage serves as a preliminary step toward replaying the trajectories on the real robot, while also enabling an assessment of the influence of finger ab/adduction. This validation consists of replaying the PCA + ProMP mean trajectories on robots to verify that the synthesized motions are executable and coherent under realistic constraints.

We begin with a simulation using the Shadow Hand, a 24-DOF humanoid robotic hand of which 20 DOFs are used in this study. For each of the four fingers of the robotic hand, MCP, PIP, and DIP flexion/extension plus abduction/adduction are available. For the thumb, CMC, MCP, and IP flexion/extension as well as thumb abduction/adduction are modeled. Because the recorded kinematic DOFs match the robot’s controllable DOFs one-to-one, we retarget trajectories via direct joint-to-joint mapping, with unit conversion, offset correction, and saturation to the robot’s joint limits.

On this platform, we execute, under different conditions, the 20-DOF joint-space trajectories obtained by taking the task-specific ProMP mean in the PCA space and mapping it back to joint space via the inverse PCA transform.

**FIGURE 11:**
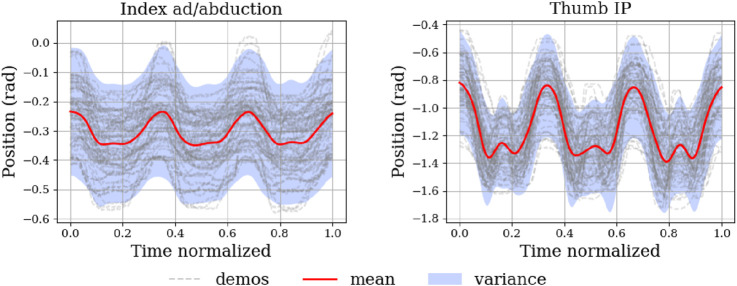
The task of an obstacle cleaned with the thumb. The ProMP-mean trajectory is shown in red and its variance in blue. These means and variances are built from the recorded trajectories, which appear in grey. ProMPs reproduce the overall shape of the training trajectories. Occasional demonstrations that fall outside the plotted variance are expected due to variability of the trials, or measurement noise.

From a task point of view, the simulation does not include an explicit model of dirt or surface cleanliness. Instead, we rely on kinematic criteria that correspond to an intuitive notion of a cleaning motion: move in the intended wiping direction, and cover approximately the same contact region as in the human demonstrations. For all five training tasks, visual inspection of the simulated executions and the contact-state traces indicates that the Shadow Hand makes contact with the virtual table or obstacle and broadly follows the demonstrated wiping path. In this sense, at the level of finger kinematics, the robot performs the same type of cleaning movements as the humans. The question of whether these motions effectively clean a physical surface is then addressed on the real robot in the next subsection, where we evaluate force control, coverage, and repeatability. For each of the five training tasks, the virtual Shadow Hand is placed in contact with a simulated table or obstacle geometry that matches the human demonstrations (flat surface, table edge, corner, or sunglasses). The ProMP-generated joint trajectories are then executed as cleaning motions on this surface, so that the hand maintains sliding contact while following the same direction of motion and contact phases as in the recorded human trials. An overview is provided in Figure 11, where ProMP-mean trajectories are plotted in red and the corresponding variances are shown in blue. As expected for a probabilistic model, the mean trajectory lies centrally within the demonstrated original trajectories and follows their key movement phases. These key phases are precisely what we aim to reproduce on the robot. Accordingly, we replay the mean trajectory on the platform and verify that the same phases emerge during execution. First, we deploy the five task-specific ProMPs and replay them without any modifications on the robot. Overall, the key movement phases observed during execution closely match those of the learned trajectories, which is consistent with the low MSE values reported earlier. Beyond nominal replay, we evaluate the model’s generalization capabilities by deliberately modifying execution conditions. In particular, we exploit two standard modulation mechanisms of movement-primitive frameworks: the via-point conditioning and the temporal retiming to adapt the same learned primitive to new situations.

In our probabilistic setting, a via-point is enforced by conditioning the primitive so that the low-dimensional state attains a specified target at a chosen time instant (i.e., constraining the trajectory to pass through a desired configuration without re-training the model). This operation alters the distribution of trajectories in a principled way while preserving their overall structure.

**FIGURE 12:**
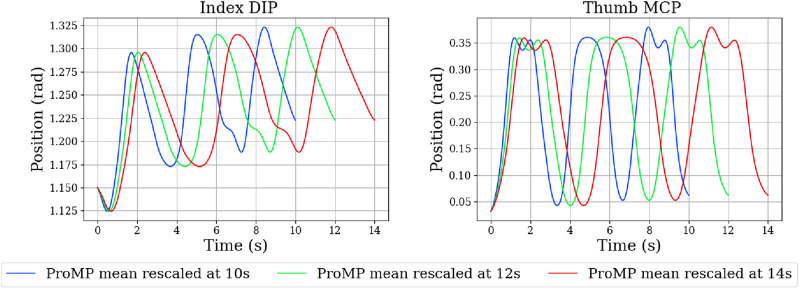
Influence of the ProMP’s temporal scaling factor on the execution of a single trajectory.

**FIGURE 13:**
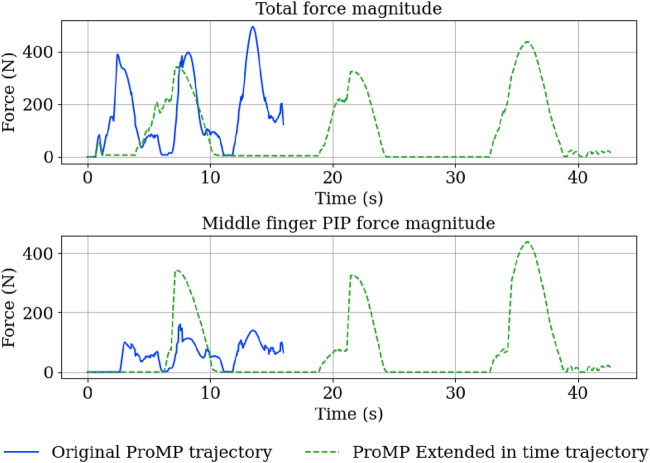
Force applied on the surface for two executions of the same cleaning trajectory with different temporal scaling factors. The contact-force profiles illustrate that time rescaling shifts the timing of the phases. For visualization, the signals were smoothed with a simple moving-average filter (rectangular window, window length = 30 samples).

**FIGURE 14:**
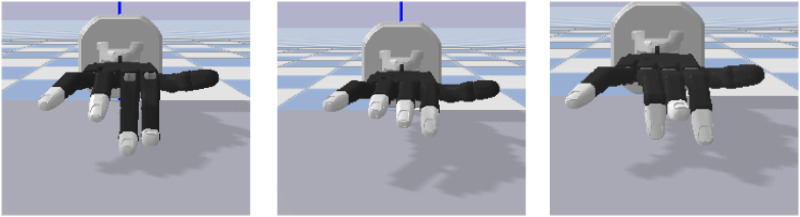
Hand normal position in the middle and passing through the via-points for the left and right graph. For the figure on the right, we play with the joint linked to the adductions of the ring, index and middle fingers.

**FIGURE 15:**
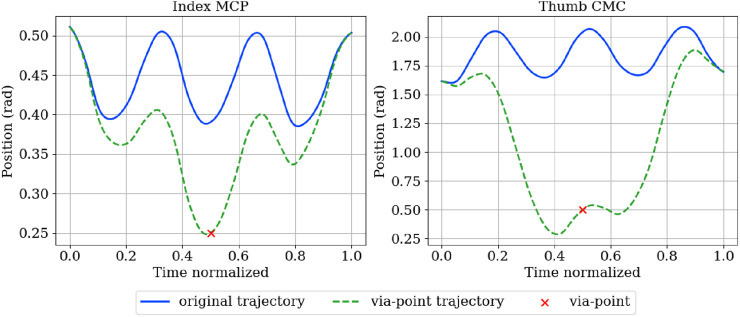
For the sunglasses task, the ProMP baseline trajectory is shown in blue and the via-point–conditioned trajectory in green. The via-points are indicated by red crosses.

We first demonstrate temporal retiming. In Figure 12, the same trajectory is executed with different temporal scalings. In Figure 13, the plots report index-finger contact force against a simulated table, showing that the intended phases occur at shifted times yet the spatial profile of the motion is maintained. We then illustrate spatial conditioning using via-points (Figures 14, 15): at a specified time *t*, selected joints are constrained to pass through target positions, while the remaining joints follow the nominal plan. The resulting executions meet the imposed constraints without distorting unrelated coordinates, indicating that the model supports localized spatial adjustments while maintaining overall trajectory coherence.

We define via-points on a subset of synergies, namely, those that contribute most to the joints that are directly involved in the adaptation to some task. We do not claim that this is the only or the optimal way to exploit ProMP conditioning, rather, we view it as a straightforward and interpretable choice that is sufficient for the proof-of-concept experiments presented here.

### 4.4 Experiment on the real robot

Consistent with the simulation, task trajectories are taken directly from the mean trajectories of the five task-specific ProMPs learned for the five cleaning tasks and retargeted to the robot via Cartesian mapping. For each task, we generate four trajectory variants by modifying via-points (spatial conditioning) and execution speed (temporal retiming). In the sunglasses condition, the manipulated factor was surface thickness. For the remaining tasks, we modulate the surface tilt and/or the surface location. In total, we evaluate 40 trajectories (5 tasks × 4 variants), each repeated three times on the robot.

Because standardized qualitative or quantitative criteria for evaluating dexterous contact manipulation are not yet established, we adapt evaluation principles from the robotic grasping literature (Howard and Kumar, 1996; Starke and Asfour, 2024). Our assessment considered four criteria:.

**FIGURE 16:**
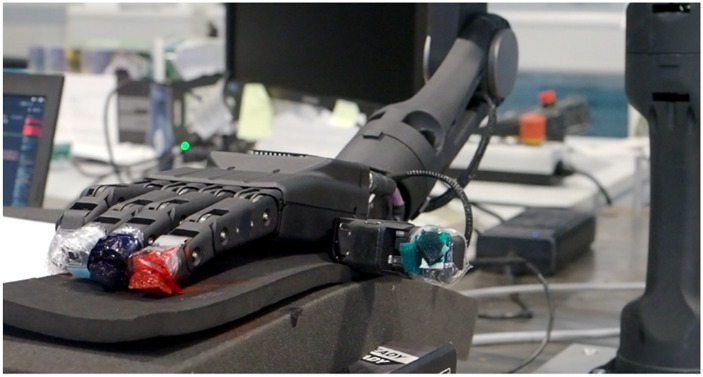
Handeffector in execution with paint at the fingertip for the Precision aspect.

- Force performance quantifies the ability to maintain a consistent contact force on the cleaning surface using the hand’s force-feedback sensors.
- Precision is evaluated by applying ink (Figure 16) to the fingertips and verifying the overlap of contact traces on the surface across the four trajectory variants.
- Representativeness captures whether the robot’s motion faithfully reproduces the original human cleaning trajectory (visual assessment).
- Repeatability measures the consistency of execution across the three repetitions.

From a human perspective, a cleaning movement is typically judged using a few intuitive criteria: whether contact with the surface is maintained, the normal force is sufficient and reasonably stable, the relevant area is covered, and the motion is reproducible.

Our four criteria mirror these aspects: Force performance captures the normal-force requirement, Precision and Representativeness together address contact continuity and surface coverage, and Repeatability quantifies how consistently the motion can be reproduced across trials. We do not directly quantify how clean the surface becomes (amount of dirt removed). Instead, we apply ink to the fingertips to visualize where and how consistently contact occurs. Taken together, these criteria allow us to assess whether the robot performs plausible cleaning motions in the selected tasks, in a way that is consistent with how human cleaning performance is usually assessed in ergonomic and robotics studies.

This evaluation protocol has also been used in our prior work and provides a consistent way to assess cleaning trajectories.

Following the point-based scoring approach used for success phases in grasping (e.g., lifting, rotating) (Starke and Asfour, 2024), we assign one point per satisfied criterion: Force Performance, Precision, Representativeness, and Repeatability. A score of 1 indicates that the execution fully meets the criterion. Otherwise, the score was 0. Aggregate scores are reported per trajectory variant and task.

Altogether, this yields 480 evaluation points (40 trajectories × 4 criteria × 3 repetitions). In total, we obtain a score of 392 out of 480, corresponding to an 81.7% success rate. Videos of the executions are available in the Supplementary Material.

### 4.5 Cross-task generalization

Beyond task-wise reconstruction, we leverage a key property of ProMPs introduced by Paraschos et al. and evaluate whether trajectories from eight held-out cleaning tasks (i.e., tasks not used to fit the ProMPs) can be recovered by the learned library. For each of these eight tasks, we consider ten held-out demonstrations.

Each test trajectory is standardized, projected into the global PCA space, and compared against three reconstructions, using the composition mechanisms introduced in Section 3.6: best-single selection of the nearest expert mean, convex blends of expert means with nonnegative weights that sum to one, and a Product-of-Experts (PoE) combination in weight space. All reconstructions are mapped back to joint space by inverse PCA, and we report joint-space MSE and RMSE.

The aggregated and per-task results are summarized in Table 5. In short, *best-single* attains the lowest errors on average (“Overall” row). *Blend* approaches *best-single* on some tasks (e.g., Bowl, Corner full hand) but is generally higher elsewhere, and *PoE* is the most conservative, with the largest errors. Practically, *blend* remains useful as a diagnostic: sparse activations indicate that the unseen trajectory lies near a small subset of experts, whereas diffuse activations with high error reveal novelty outside the current library. *PoE* is best viewed as a principled synthesis tool for co-activation under constraints rather than an inverse reconstruction method for single unseen trajectories.

**TABLE 5:** Cross-task generalization on held-out trajectories (joint-space errors). We report mean MSE and RMSE for the nearest single expert, a convex blend of expert means in PCA space, and a Product-of-Experts (uniform co-activation) in weight space. Best method is highlighted in boldface.

Composition strategy	Best-single		Blend (convex)		PoE (uniform)	
Task	MSE	RMSE	MSE	RMSE	MSE	RMSE
Curved bottle	0.251	0.499	0.320	0.558	0.478	0.675
Obstacle cleaned with 1 finger	0.066	0.255	0.257	0.495	0.308	0.544
Obstacle cleaned with the full hand	0.126	0.349	0.325	0.543	0.354	0.586
Edge of a table	0.239	0.470	0.313	0.549	0.359	0.597
Bowl	0.157	0.393	0.158	0.391	0.254	0.490
Corner of a table cleaned with 2 fingers (rotational)	0.200	0.446	0.306	0.545	0.318	0.560
Corner of a table cleaned with 4 fingers (rotational)	0.220	0.460	0.231	0.472	0.321	0.556
Dusting	0.202	0.448	0.312	0.557	0.348	0.588
Overall	**0.183**	**0.415**	0.278	0.514	0.343	0.575

## 5 Discussion

Our results indicate that a simple synergy representation, i.e., a linear PCA basis in joint space combined with task-specific ProMPs, can reproduce the main phases of contact-rich finger cleaning motions across a family of tasks. In both the 20-DOF and 15-DOF settings, the average normalized joint-space reconstruction error is around 9%, and the resulting mean trajectories remain qualitatively consistent when replayed in simulation and on the Aeon hardware, including under temporal rescaling and via-point conditioning. Overall, these findings suggest that a low-dimensional synergy-based movement-primitive interface can be practically deployed on robotic hands for related finger-cleaning skills, with a modest loss in reconstruction fidelity compared to primitives learned directly in the full joint space. From the perspective of hand synergies, the PCA analysis confirms that the recorded cleaning motions are strongly organized along a few coordinated patterns rather than independent joint control. In the 20-DOF case, the leading components capture a global flexion mode dominated by PIP/DIP activity, followed by components describing thumb posture and lateral refinements between ulnar and radial fingers, while ab/adduction emerges mainly in higher components. In the 15-DOF case (flexion/extension only), a similar structure appears without the explicit spread component. This organization is consistent with classical synergy findings in human grasping and manipulation, where a small number of postural and force synergies explain most of the variance across tasks. It also explains why reconstruction errors remain low: many of the cleaning tasks are dominated by distal flexion/extension patterns with comparatively small MCP modulation, so a handful of principal components already captures most of the reproducible kinematics. Conversely, tasks such as “curved bottle” or wiping along the table edge require more intricate, time-varying coordination of MCP, PIP, and DIP, and thus exhibit higher errors in the cross-task evaluations, in line with their higher effective dimensionality. Compared to task-space embeddings that tightly couple the representation to specific object geometries, joint-space synergies offer a robot-ready linear decoder back to joint space that can be reused across different cleaning scenarios with varying objects and surface shapes.

Because we evaluate our approach in two kinematic configurations (15-DOF flexion/extension only vs. 20-DOF including abduction/adduction), it is important to interpret how this design choice affects reconstruction. The difference between the 20-DOF and 15-DOF configurations is small and method-dependent. For ProMP-only, the 20-DOF setting yields a marginally lower average MSE than the 15-DOF setting (Table 3), whereas for PCA + ProMP the averages are very close and slightly favor the 15-DOF setting. This suggests that adding abduction/adduction increases representational capacity but also introduces variability that is only partially captured when trajectories are projected onto a reduced synergy space. We therefore do not interpret either configuration as qualitatively superior: both recover the main structure of the cleaning movements, with the 15-DOF setting remaining attractive for platforms that primarily actuate flexion/extension.

Since hand-synergy results for grasping tasks are often reported in terms of how much variance a small number of (static) eigenpostures explains, we would like to compare our cleaning reconstruction errors to those classic grasping benchmarks. However, the comparison is not direct. While an MSE of roughly 8%–9% is clearly higher than the variance explained by a single static eigenposture in classic grasping studies, the comparison is not direct. We reconstruct full time-varying trajectories in a contact-rich setting, not single postures under tightly controlled conditions. Demonstrations differ in timing, contact configuration, and detailed finger placement, especially for tasks with several equally valid strategies. In addition, our quantitative metric compares the ProMP mean trajectory to individual demonstrations, so some error is inevitable even if the model perfectly captured the underlying distribution. From this perspective, the reported errors should be interpreted as the typical deviation between individual demonstrations and the learned mean trajectory. This deviation conflates model bias and inter-demonstration variability (timing, contact configuration, and finger placement), rather than indicating a pure failure to reproduce the data.

Comparing PCA + ProMP to a ProMP trained directly in joint space highlights a trade-off.

In our dataset, learning a ProMP directly in joint space achieves slightly lower reconstruction MSE than PCA + ProMP (20-DOF: 8.58% for the ProMP method vs. 9.02% for the PCA + ProMP method, and 15-DOF: 8.65% for the proMP method vs. 8.94% for the PCA + ProMP), which is expected since PCA introduces a projection bias by restricting trajectories to 5/7 principal components. These observations indicate a better fit of the demonstrations in the full space. It also addresses the concern that ProMPs might not need dimensionality reduction. PCA nevertheless remains valuable for our objectives: it provides a shared low-dimensional synergy space that supports cross-task composition and modulation. In addition, operating in the low-dimensional synergy space substantially reduces the overall computation time in our implementation (15-DOF: 102.4 s → 13.1 s, 20-DOF: 198.8 s → 19.9 s at M=20 ). Peak memory consumption exhibits the same trend as computation time. ProMP-only consistently uses more memory than PCA + ProMP. The main reason is that the ProMP formulation involves matrix operations whose cost grows superlinearly with the trajectory dimension. When learning directly in joint space, the dimensionality of the weight vector is $w\in R^{D\cdot M}$ , so storing and manipulating quantities such as the weight covariance becomes expensive. In addition, estimating per-demonstration weights via ridge regression requires solving linear systems and may allocate sizable intermediate buffers. PCA + ProMP reduces these costs by working in a synergy space of dimension k≪D , thereby shrinking both the persistent model parameters and more importantly the largest temporary objects that dominate peak memory usage.

These results suggest that dimensionality reduction is not strictly required to obtain low reconstruction error in our setting. Rather, PCA primarily serves as a structural constraint that provides a shared latent space for cross-task composition and modulation, at the cost of a small loss in reconstruction fidelity due to projection onto 5/7 components. This efficiency-oriented perspective is supported by prior studies where movement primitives serve as structured policy representations for policy-search reinforcement learning: reducing the dimensionality of the policy (e.g., via synergies/latent variables) is a practical lever to improve sample efficiency and make on-robot optimization feasible (Colomé and Torras, 2018; Colomé et al., 2014). In that sense, even when joint-space ProMPs provide slightly better MSE, the PCA + ProMP formulation remains attractive as a compact, shared latent space that enables composition and modulation across tasks with lower computational cost. Furthermore, beyond learning, dimentionality reduction techniques provide a low-dimensional control interface: modulating a few latent coefficients can yield smoother and more stable finger coordination than acting directly in the full joint space, which is advantageous for contact-driven execution.

Regarding cross-task generalization, the experiments with the eight held-out cleaning tasks show that a small library of five ProMPs learned from the training tasks already covers a meaningful portion of the remaining motions. In the PCA space, selecting the single expert whose mean trajectory is closest to a held-out coefficient curve yields the lowest reconstruction errors on average, indicating that several unseen tasks are essentially variants of one learned prototype. Convex blends of expert means further extend the coverage by linearly composing synergies, and the structure of the weights provides a diagnostic of whether an unseen motion lies near the convex hull of the library (sparse weights) or outside it (diffuse weights and higher errors). The Product-of-Experts co-activation remains the most conservative mechanism, as expected, and is better interpreted as a principled tool for enforcing constraints (e.g., via-points) than as an inverse reconstruction method. Altogether, these results support the view that a small set of synergy-based ProMPs can serve as a reusable skill library from which new cleaning motions can be approximated by selection or blending in the latent space.

In terms of robotic cleaning, the behaviour observed on the Shadow Hand and the Aeon hand suggests several practical implications. First, using a low-dimensional synergy basis to parameterize ProMPs makes it possible to represent a family of fine-grained cleaning skills with a compact set of parameters that are easy to interpret and to store. Rather than authoring or teleoperating a new joint-space trajectory for every object and surface, a robot could reuse a small library of ProMPs and adjust them through temporal scaling and via-point conditioning to accommodate changes in surface pose, reachability, or obstacle placement. Second, working in joint space with a simple inverse PCA mapping allows the synergy trajectories to be combined with existing arm or whole-body motion planners: a high-level planner positions the hand relative to the environment, while the PCA + ProMP controller regulates local contact at the fingers. Third, the real-robot experiments on the Aeon hand, with an overall success rate of about 82% across force performance, precision, representativeness, and repeatability, show that the learned primitives do not only work in simulation but withstand calibration offsets, compliance, and sensing noise in hardware. In the majority of trials, the executed motions maintained stable contact, applied reasonable forces, and followed the intended contact path on the surface.

Although the combination of PCA and ProMPs we use is not algorithmically novel, our contribution lies in applying this classical synergy-based framework to a regime that has received little attention so far: continuous, contact-rich cleaning motions at the finger-joint level, executed on both a simulated 20-DOF hand and a physical humanoid hand. Our results show that this relatively simple representation is already sufficient to capture and reproduce non-trivial multi-finger coordination, and to support compositional generalization across a family of related cleaning tasks. This suggests that well-understood linear synergies combined with probabilistic movement primitives remain a practical and effective option for controlling dexterous robot hands in in-hand–like manipulation scenarios, without resorting to heavier models. This makes the approach interesting as a practical control layer for in-hand–like cleaning skills, where having a stable, low-dimensional interface to the hand is often more valuable than algorithmic novelty.

At the same time, several important limitations must be acknowledged. First, our representation is purely kinematic: we do not explicitly model forces, friction, or tactile feedback, and the ProMPs are not adapted online based on contact information. As a consequence, robustness under large variations in surface friction, compliance, or external disturbances remains to be demonstrated. Second, although our acquisition setup captured full upper-limb kinematics with an optical motion-capture system, the present study explicitly restricts learning and control to finger-joint trajectories. In practice, however, cleaning behaviours are produced by the coupled motion of arm, wrist, and hand: the arm largely determines the global path and orientation of the contact patch on the surface, while the fingers regulate local contact geometry and pressure. For applications that require reasoning about the total wrench applied to the environment (for example, scrubbing with substantial normal forces or long strokes across the workspace), it would therefore be desirable to extend our approach to a joint arm–hand representation. One promising direction is to learn movement primitives in which a global arm primitive specifies the end-effector path and orientation, and a local hand ProMP, expressed in the synergy space introduced here, controls the fine-scale contact modulation at the fingers. Developing such coupled arm–hand models, and assessing their benefits for force-controlled, full-stroke cleaning, is an important objective for future work. Third, generalization is evaluated within a single class of tasks (table- and object-cleaning) and under moderate variations in geometry, and we do not compare against nonlinear dimensionality-reduction methods or alternative primitive formulations under matched conditions. Exploring alternative synergy spaces (e.g., nonlinear embeddings) while preserving interpretability and an invertible mapping to joint space is an interesting direction for future work. Fourth, the current framework for the Shadow hand relies on a simple calibrated joint-to-joint mapping between the SenseGlove and the Shadow hand, which in terms of design is the closest to the human hand. This choice makes retargeting straightforward, but it ignores task-space structure. More advanced Cartesian or hybrid schemes that explicitly align fingertip poses and contact locations may better exploit the robot hardware. Nevertheless, our experiments indicate that this baseline is sufficient to obtain coherent cleaning motions and reproduce the main contact phases. Finally, our main quantitative metrics are based on reconstruction error and cross-task MSE/RMSE, more probabilistic evaluations (e.g., likelihood of demonstrations under the learned ProMPs) and formal comparisons to additional baselines would further strengthen the analysis. Future work will therefore focus on integrating contact and force feedback into the synergy-based ProMPs, extending the approach to more diverse manipulation scenarios, and exploring richer synergy spaces while preserving the interpretability and hardware compatibility that motivated the present design. We also plan to investigate alternative finger-level mapping strategies and quantify their impact on force control, surface coverage, and cross-task generalization.

## Data Availability

The raw data supporting the conclusions of this article will be made available by the authors, without undue reservation.

## References

[B1] Amor H. B. Kroemer O. Hillenbrand U. Neumann G. Peters J. (2012). “Generalization of human grasping for multi-fingered robot hands,” in Proceedings of the 2012 IEEE/RSJ International Conference on Intelligent Robots and Systems (IROS) (IEEE), 2043–2050.

[B2] Bitzer S. Vijayakumar S. (2009). “Latent spaces for dynamic movement primitives,” in Proceedings of the IEEE-RAS International Conference on Humanoid Robots (Humanoids). 10.1109/ICHR.2009.5379530

[B3] Brahmbhatt S. Ham C. Kemp C. C. Hays J. (2019). “Contactdb: analyzing and predicting grasp contact via thermal imaging,” in IEEE/CVF Conference On Computer Vision and Pattern Recognition (CVPR).

[B4] Calinon S. (2013). “On improving the extrapolation capability of task-parameterized movement models,” in Proceedings of the IEEE/RSJ International Conference on Intelligent Robots and Systems (IROS).

[B5] Cohen Y. Bar-Shira O. Berman S. (2021). Motion adaptation based on learning the manifold of task and dynamic movement primitive parameters. Robotica 39, 1299–1315. 10.1017/s0263574720001186

[B6] Colomé A. Torras C. (2018). Dimensionality reduction for dynamic movement primitives and application to bimanual manipulation of clothes. IEEE Trans. Robotics 34, 602–615. 10.1109/TRO.2018.2808924

[B7] Colomé A. Neumann G. Peters J. Torras C. (2014). “Dimensionality reduction for probabilistic movement primitives,” in 2014 IEEE-RAS International Conference on Humanoid Robots (IEEE), 794–800.

[B8] Cushion E. J. Warmenhoven J. North J. S. Cleather D. J. (2019). Principal component analysis reveals the proximal to distal pattern in vertical jumping is governed by two functional degrees of freedom. Front. Bioeng. Biotechnol. 7, 193. 10.3389/fbioe.2019.00193 31440505 PMC6694595

[B9] Daffertshofer A. Lamoth C. C. J. Meijer O. G. Beek P. J. (2004). Pca in studying coordination and variability: a tutorial. Clin. Biomech. 19, 415–428. 10.1016/j.clinbiomech.2004.01.005 15109763

[B10] Deluzio K. J. Astephen J. L. (2007). Biomechanical features of gait waveform data associated with knee osteoarthritis: an application of principal component analysis. Gait and Posture 25, 86–93. 10.1016/j.gaitpost.2006.01.007 16567093

[B11] Fan Z. Taheri O. Tzionas D. Kocabas M. Kaufmann M. Black M. J. (2023). “Arctic: a dataset for dexterous bimanual hand-object manipulation,” in IEEE/CVF conference on computer vision and pattern recognition (CVPR).

[B12] Feix T. Romero J. Schmiedmayer H. Dollar A. M. Kragic D. (2015). The grasp taxonomy of human grasp types. IEEE Trans. Human-Machine Syst. 46, 66–77. 10.1109/thms.2015.2470657

[B13] Fu R. Zhang D. Jiang A. Fu W. Funk A. Ritchie D. (2025). Gigahands: a massive annotated dataset of bimanual hand activities. Prepr. CVPR 2025 Highlight. 10.1109/CVPR52734.2025.01627

[B14] Gao T. Nasiriany S. Liu H. Yang Q. Zhu Y. (2024). Prime: scaffolding manipulation tasks with behavior primitives for data-efficient imitation learning. IEEE Robotics Automation Lett. 10.48550/arXiv.2403.00929

[B15] Gioioso G. Salvietti G. Malvezzi M. Prattichizzo D. (2013). Mapping synergies from human to robotic hands with dissimilar kinematics: an approach in the object domain. IEEE Trans. Robotics 29, 825–837. 10.1109/tro.2013.2252251

[B16] Howard W. S. Kumar V. (1996). On the stability of grasped objects. IEEE Trans. Robotics Automation 12, 904–917. 10.1109/70.544773

[B17] Iberall T. (1986). The representation of objects for grasping. Proc. Annu. Meet. Cognitive Sci. Soc. 8.

[B18] Ijspeert A. J. Nakanishi J. Hoffmann H. Pastor P. Schaal S. (2013). Dynamical movement primitives: learning attractor models for motor behaviors. Neural Comput. 25, 328–373. 10.1162/NECO_a_00393 23148415

[B19] Jolliffe I. (2011). “Principal component analysis,” in International Encyclopedia of Statistical Science (Springer), 1094–1096.

[B20] Jolliffe I. T. Cadima J. (2016). Principal component analysis: a review and recent developments. Philosophical Transactions Royal Society A Math. Phys. Eng. Sci. 374, 20150202. 10.1098/rsta.2015.0202 26953178 PMC4792409

[B21] Kim J. Cauli N. Vicente P. Damas B. Bernardino A. Santos-Victor J. (2020). Cleaning tasks knowledge transfer between heterogeneous robots: a deep learning approach. J. Intelligent and Robotic Syst. 98, 191–205. 10.1007/s10846-019-01072-4

[B23] Krug R. Dimitrov D. (2015). Model predictive motion control based on generalized dynamical movement primitives. J. Intelligent and Robotic Syst. 77, 17–35. 10.1007/s10846-014-0100-3

[B22] Krebs F. Meixner A. Patzer I. Asfour T. (2021). The kit bimanual manipulation dataset. Tech. Report, KIT Whole-Body Hum. Motion Database. 10.1109/HUMANOIDS47582.2021.9555788

[B25] Li R. Wang H. Liu Z. (2021). Survey on mapping human hand motion to robotic hands for teleoperation. IEEE Trans. Circuits Syst. Video Technol. 32, 2647–2665. 10.1109/tcsvt.2021.3057992

[B24] Lew T. Singh S. Prats M. Bingham J. Weisz J. Holson B. (2022). Robotic Table Wiping via Reinforcement Learning and Whole-Body Trajectory Optimization. Preprint.

[B26] Li W. Chen G. Zhao T. Wang J. Hu T. Liao Y. (2025). Cleanupbench: Embodied Sweeping and Grasping Benchmark. Preprint.

[B27] Liarokapis M. V. Artemiadis P. K. Kyriakopoulos K. J. (2013). “Mapping human to robot motion with functional anthropomorphism for teleoperation and telemanipulation with robot arm hand systems,” in 2013 IEEE/RSJ International Conference on Intelligent Robots and Systems (IEEE), 2075.

[B28] Mason C. R. Gomez J. E. Ebner T. J. (2001). Hand synergies during reach-to-grasp. J. Neurophysiology. 10.1152/jn.2001.86.6.2896 11731546

[B29] Matsubara T. Hyon S.-H. Morimoto J. (2010). “Learning stylistic dynamic movement primitives from multiple demonstrations,” in IROS. 10.1016/j.neunet.2011.02.004 21388784

[B30] Paraschos A. Daniel C. Peters J. R. Neumann G. (2013). Probabilistic movement primitives. Adv. Neural Inf. Process. Syst. (NeurIPS) 26.

[B31] Paraschos A. Daniel C. Peters J. Neumann G. (2018a). Using probabilistic movement primitives in robotics. Aut. Robots 42, 529–551. 10.1007/s10514-017-9648-7

[B32] Paraschos A. Rueckert E. Peters J. Neumann G. (2018b). Probabilistic movement primitives under unknown system dynamics. Adv. Robot. 32, 297–310. 10.1080/01691864.2018.1437674

[B33] Patel V. Burns M. Mao Z.-H. Crone N. E. Vinjamuri R. (2015). Linear and nonlinear kinematic synergies in the grasping hand. J. Bioeng. and Biomed. Sci. 5 (1). 10.4172/2155-9538.1000163

[B34] Pignat E. Calinon S. (2017). Learning adaptive dressing assistance from human demonstration. Robotics Aut. Syst. 93, 61–75. 10.1016/j.robot.2017.03.017

[B35] Rueckert E. Mundo J. Paraschos A. Peters J. Neumann G. (2015). “Extracting low-dimensional control variables for movement primitives,” in ICRA.

[B36] Santello M. Soechting J. F. (2000). Force synergies for multifingered grasping. Exp. Brain Res. 10.1007/s002210000420 10985681

[B37] Santello M. Flanders M. Soechting J. F. (1998). Postural hand synergies for tool use. J. Neuroscience 18, 10105–10115. 10.1523/JNEUROSCI.18-23-10105.1998 9822764 PMC6793309

[B38] Saudabayev A. Rysbek Z. Khassenova R. Varol H. A. (2018). Human grasping database for activities of daily living with depth, color and kinematic data streams. Sci. Data 5, 180101. 10.1038/sdata.2018.101 29809171 PMC5972673

[B39] Saveriano M. Abu-Dakka F. J. Kramberger A. Peternel L. (2023a). Dynamic movement primitives in robotics: a tutorial survey. Int. J. Robotics Res. 42, 1133–1184. 10.1177/02783649231201196

[B40] Saveriano M. Abu-Dakka F. J. Kyrki V. (2023b). Learning stable robotic skills on riemannian manifolds. Robotics Aut. Syst. 169, 104510. 10.1016/j.robot.2023.104510

[B41] Schaal S. (2006). “Dynamic movement primitives–a framework for motor control in humans and humanoid robotics,” in Adaptive Motion of Animals and Machines. Editors Hirose S. Morishima K. (Springer), 261–280.

[B42] SenseGlove B. V. (2025). “Senseglove_ros: ros interfaces for senseglove,” in GitHub organization: Adjuvo. Maintainer: Akshay R. Menon. Commit USED: e5e9a58. Available online at: https://github.com/Adjuvo/senseglove_ros (Accessed December 2, 2025).

[B43] Starke J. Asfour T. (2024). “Kinematic synergy primitives for human-like grasp motion generation,” in Proceedings of the 2024 IEEE International Conference on Robotics and Automation (ICRA) (IEEE), 4119–4125.

[B44] Taheri O. Ghorbani N. Black M. J. Tzionas D. (2020). “Grab: a dataset of whole-body human grasping of objects,” in European Conference on Computer Vision (ECCV).

[B45] van der Maaten L. J. P. Postma E. O. van den Herik H. J. (2009). Dimensionality reduction: a comparative review. J. Machine Learning Research 10, 13.

[B46] Zhan X. Yang L. Zhao Y. Mao K. Xu H. Lin Z. (2024). “Oakink2: a dataset of bimanual hands-object manipulation in complex task completion,” in IEEE/CVF Conference on Computer Vision and Pattern Recognition (CVPR).

[B47] Zhao T. Deng M. Li Z. Hu Y. (2020). Cooperative manipulation for a mobile dual-arm robot using sequences of dynamic movement primitives. IEEE Trans. Cognitive Dev. Syst. 12, 18–29. 10.1109/TCDS.2018.2868921

